# Enhancement of Ionization Efficiency Using Zeolite in Matrix-Assisted Laser Desorption/Ionization Mass Spectrometry of Multiple Drugs in Cancer Cells (Mass Spectrometry of Multiple Drugs in Cells Using Zeolite)

**DOI:** 10.5702/massspectrometry.A0091

**Published:** 2020-12-04

**Authors:** Hiroki Kannen, Shusei Nomura, Hisanao Hazama, Yasufumi Kaneda, Tatsuya Fujino, Kunio Awazu

**Affiliations:** 1 Graduate School of Engineering, Osaka University, 2–1 Yamadaoka, Suita, Osaka 565–0871, Japan; 2 Graduate School of Medicine, Osaka University, 2–2 Yamadaoka, Suita, Osaka 565–0871, Japan; 3 Department of Applied Chemistry, Graduate School of Science and Engineering, Toyo University, 2100 Kujirai, Kawagoe, Saitama 350–8585, Japan; 4 Graduate School of Frontier Biosciences, Osaka University, 1–3 Yamadaoka, Suita, Osaka 565–0871, Japan; 5 Global Center for Medical Engineering and Informatics, Osaka University, 2–2 Yamadaoka, Suita, Osaka 565–0871, Japan

**Keywords:** matrix-assisted laser desorption/ionization, zeolite matrix, anticancer drug, photodynamic therapy, multiple drugs

## Abstract

Combined therapy using photodynamic therapy (PDT) and chemotherapy has been proposed for anticancer-drug-resistant cancer cells. To evaluate the efficacy of such a combined therapy, the uptakes of an anticancer drug and a photosensitizer in cancer cells must be assessed. Mass spectrometry using matrix-assisted laser desorption/ionization can detect multiple drugs simultaneously. Human prostate cancer cells PC-3 or docetaxel-resistant cancer cells PC-3-DR were incubated in a serum-free medium containing a photosensitizer, protoporphyrin IX (PpIX), and an anticancer drug, docetaxel. A zeolite matrix was created by mixing 6-aza-2-thiothymine and NaY5.6 zeolite, and dissolving in water with 50% acetone. Ions were obtained with a time-of-flight mass spectrometer using a Nd:YAG laser at a wavelength of 355 nm. The cell morphology was preserved by washing the cells with ammonium acetate and drying in a vacuum after drug administration. Protonated PpIX (*m*/*z* 563.3) and the sodium adduct ion of docetaxel (*m*/*z* 829.9) were obtained from PC-3 cells simultaneously using the zeolite matrix. On the other hand, PpIX was detected but ions originating from docetaxel were not detected from PC-3-DR cells. The result indicated the efficacy of PDT for docetaxel-resistant cancer cells.

## INTRODUCTION

In chemotherapy, the therapeutic effect decreases with time due to the existence of anticancer-drug-resistant cancer cells. For example, docetaxel is often used to treat prostate cancers, but the response rate of docetaxel is low (44.2% when used with prednisolone),^1)^ and drug-resistant cells, which are refractory to treatment, increase over time. These cancer cells have P-gps (permeability-glycoprotein) on the plasma membrane, which has a high ability to excrete extracellular compounds such as cytotoxic compounds. The excretion makes accumulation of docetaxel difficult and reduces the efficacy of the therapeutic.^2–4)^

Recently, a combined therapy using photodynamic therapy (PDT) and chemotherapy has been suggested to improve the efficacy of treatment.^5–7)^ In PDT, a photosensitizer selectively accumulates in the tumor tissue inside a patient’s body, and then reactive oxygen species, which are generated by irradiating with a laser or another light source, target cancer cells. An advantage of PDT is that it is a less invasive therapy for normal tissue. To evaluate the efficacy of such a combined therapy, it is necessary to assess the uptakes of an anticancer drug and a photosensitizer in cancer cells.

To assess the uptakes of multiple drugs in cancer cells, our research employs matrix-assisted laser desorption/ionization mass spectrometry (MALDI-MS). MALDI-MS can detect multiple drugs without labeling.^8)^ However, the major disadvantage of MALDI-MS is that its detection sensitivity depends on the sample preparation method, and the matrix is one factor that greatly affects the detection sensitivity.

To enhance the detection sensitivity, this study focuses on the zeolite matrix, which is formed by mixing a conventional organic matrix and zeolite. A zeolite matrix has been reported to increase the detection sensitivity compared with a conventional matrix.^9,10)^ Zeolite has many pores in its crystal structure, and an organic matrix is adsorbed in the pores by mixing the two. An organic matrix can maintain a stable state because the extra internal energy with the ion of an organic matrix is transferred to zeolite.

In this study, we investigate the simultaneous detection of an anticancer drug and a photosensitizer administered in cancer cells using the zeolite matrix to assess their uptakes in cancer cells. First, the efficacy of a zeolite matrix is investigated by comparing the detection sensitivity with a pure mixture of an anticancer drug and a photosensitizer. Then to assess the efficacy of the zeolite matrix, these drugs are administered in cancer cells, and the detection sensitivity of two drugs is examined using the zeolite matrix. Finally, the uptakes of multiple drugs in prostate cancer cells and its docetaxel-resistant cancer cells are evaluated.

## EXPERIMENTAL

### Time-of-flight mass spectrometer

All experiments were performed using a time-of-flight (TOF) mass spectrometer (Voyager DE-PRO, Applied Biosystems, CA, USA) equipped with a 355-nm third-harmonic Nd:YAG laser (GAIA II 30-T, Rayture Systems, Tokyo, Japan). The instrument parameters in the reflectron-mode of Voyager DE-PRO were as follows: +20 kV accelerating voltage, +13.6 kV voltage for the extraction grid, 0 V for the guide wire, and 100 ns extraction delay time.

### Sample preparation

Docetaxel, which is known by the trade name of Taxotere (Sanofi-Aventis, Paris, France), is frequently used to treat prostate cancer. In MALDI, the additive polysorbate80 in Taxotere suppresses the ionization of docetaxel.^11)^ In this study, docetaxel trihydrate without polysorbate80 was purchased from Wako (Osaka, Japan). Docetaxel hydrate dissociates in water, methanol, dimethyl sulfoxide, and in the body, and behaves as docetaxel. Protoporphyrin IX (PpIX, P8293, SIGMA-Aldrich, Tokyo, Japan) was used as a photosensitizer. PpIX is the basic skeleton of hemoglobin. Although it is metabolized in normal tissue, it accumulates in cancer cells due to the metabolic abnormality.^12)^

The organic matrix was 6-aza-2-thiothymine (ATT, 275514, SIGMA-Aldrich, Tokyo, Japan). The zeolite was sodium-substituted Y-type zeolite (NaY5.6 zeolite). ATT and NaY5.6 zeolite were mixed with the equal weights in a pestle for 10 min, and an ionization-assistant reagent, zeolite matrix (ATT/NaY), was produced. We previously reported that the signal intensity from a pure docetaxel sample at a concentration of 100 μM increased about 13-fold using a zeolite matrix with the droplet method.^13)^

In this study, we investigated two types of matrix application methods, the spray method and the droplet method. The matrix was sprayed using an airbrush (PS-153: 3500 PRO SPRAY Mk-2, Mr. Hobby, GSI Creos, Tokyo, Japan). Since the acidity of the matrix may degrade the metal nozzle and reduce the signal intensities of interest, the nozzle was replaced with a microchip (DIAMOND TIP, D10, Gilson, Middleton, WI, USA) and a glass tube (Calibrated Pipets, 2-000-001-90, Drummond Scientific, Broomall, PA, USA). Samples were held on the side of a draft at a height of about 20 cm, and the matrix was sprayed at a distance of about 20 cm between the sample and the airbrush. To prevent the immediate formation of large droplets, the spraying began at least 20 cm away from the sample. The density of the matrix coating was calculated from the unmasked area and the weight difference before and after coating.

In all experiments, an indium tin oxide (ITO)-coated glass slide (# 237001, Bruker, Billerica, MA, USA) was used as the sample plate. The signal intensity was measured by the peak height of the ion.

### Cell cultures

The cell lines were the hormone-antagonistic human prostate cancer cell line PC-3 and its docetaxel-resistant cancer cells PC-3-DR. Cells were cultured in Dulbecco’s modified Eagle’s medium (D-MEM, D6046, SIGMA-Aldrich, Tokyo, Japan) containing 10% fetal bovine serum (FBS, S1820, Biowest SAS, Nuaillé, France) and 100 units/mL each of penicillin and streptomycin (P4458, SIGMA-Aldrich, Tokyo, Japan). Cells were incubated at 37°C in an atmosphere containing 5% CO_2_. To initially acquire docetaxel resistance in PC-3-DR, docetaxel was dissolved in a medium without serum, and PC-3 was cultured for 1 h. Then the medium was replaced with a docetaxel-free medium and surviving cells were cultured in the usual method described above. After 1–2 weeks, the growth rate of the cells returned to the same as before drug administration. In this study, by repeating this method, the docetaxel resistance of PC-3-DR was increased at a concentration of 50 μM.

## RESULTS AND DISCUSSION

### Detection of multiple drugs in a pure mixture

To investigate the efficacy of the zeolite matrix for the ionization of multiple drugs, PpIX and docetaxel, we measured the drugs in a pure mixture. A pure mixture was prepared by PpIX and docetaxel in water with 50% methanol at concentrations of 5 and 30 μM, respectively. The zeolite matrix ATT/NaY was made by dissolving in water with 50% acetone at a concentration of 5 mg/mL. A pure mixture with a volume of 1 μL was dropped onto an ITO-coated glass slide and dried in a vacuum. ATT/NaY was sprayed on the dried spot of the mixture at a density of 0.070 mg/cm^2^. A laser was randomly irradiated at 10 points for one spot and 100 pulses per point. Then one mass spectrum was obtained by averaging the respective mass spectra.

**Figure 1:**
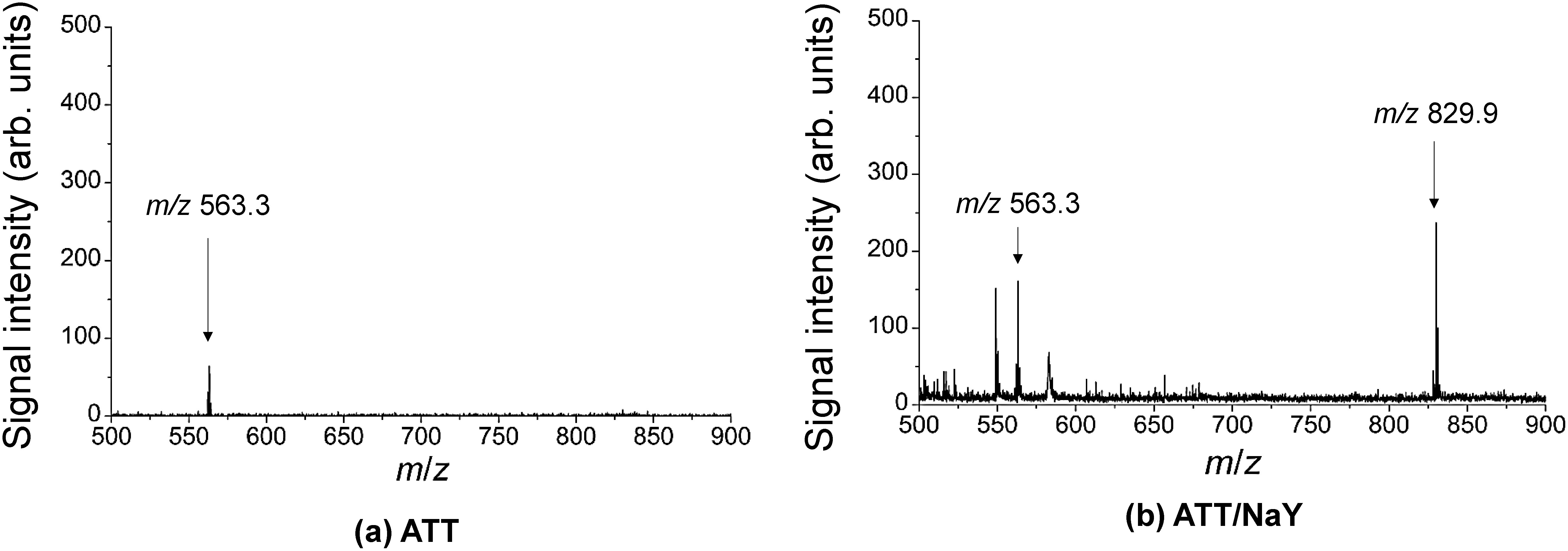
Fig. 1. Typical mass spectra obtained from a pure mixture of PpIX and docetaxel at concentrations of 5 and 30 μM with (a) ATT and (b) ATT/NaY. Each matrix is sprayed on the dried spot of the mixture.

Figure 1 shows the typical mass spectra obtained from a pure mixture of PpIX and docetaxel with (a) ATT and (b) ATT/NaY, which were sprayed on a dried spot of the mixture. The product ions for PpIX at *m*/*z* 563.3 and docetaxel at *m*/*z* 829.9 were detected using ATT/NaY. In previous studies, ion suppression was observed as the decrease in the signal intensity of the ion at *m*/*z* 829.9. Consequently, the ion of PpIX and docetaxel administered in cancer cells could not be detected simultaneously. To detect multiple drugs simultaneously, it is important that the signal intensities of drugs are at the same level. In the next experiment, PpIX and docetaxel were administered in cancer cells at concentrations of 5 and 30 μM, respectively, to detect multiple drugs in a cell suspension.

**Figure 2:**
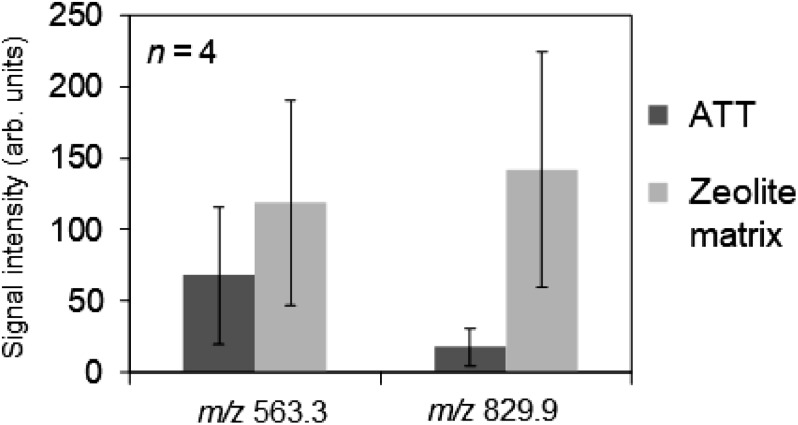
Fig. 2. Average signal intensities of the ions at *m*/*z* 563.3 and 829.9 obtained from a pure mixture of PpIX and docetaxel. ATT and ATT/NaY were sprayed on the dried spot of the mixture.

Figure 2 shows the average signal intensities of the ions at *m*/*z* 563.3 and 829.9 obtained from a pure mixture of PpIX and docetaxel when ATT and ATT/NaY were sprayed on a dried spot of the mixture. Compared to ATT only, the detection sensitivities of the ions at *m*/*z* 563.3 and 829.9 respectively increased 1.5-fold and 6.4-fold when a zeolite matrix was used with ATT. We focused on sodium-substituted Y-type zeolite to enhance the signal intensity of the sodium adduct ion for docetaxel at *m*/*z* 829.9. Not only the signal intensity of the ion at *m*/*z* 829.9 but also the signal intensity of the ion at *m*/*z* 563.3 increased. In the previous study, the signal intensity from a pure docetaxel sample increased about 13-fold using a zeolite matrix with the droplet method. The difference in signal intensities was attributed to the coating method of the matrix.

### Detection of multiple drugs in a cell suspension

To detect the drugs administered in cells simultaneously, we measured a cell suspension of PC-3. PpIX and docetaxel were dissolved in dimethyl sulfoxide (DMSO, D4540, SIGMA-Aldrich, Tokyo, Japan). The solution of PpIX and docetaxel was diluted 100-fold in the medium at concentrations of 5 and 30 μM, respectively. After PC-3 cells were cultured in a flask with a bottom area of 25 cm^2^ for 24 h, PC-3 was incubated for 1 h in a culture medium containing PpIX and docetaxel diluted as described above. As a control, PC-3 was incubated in a medium without drugs in a similar manner. After removing the medium, the cells were washed once with Dulbecco’s phosphate buffered saline (D-PBS, D8537, SIGMA-Aldrich), trypsinized, resuspended in D-PBS, and centrifuged. After removing the supernatant, the cells were dissolved in distilled water at a number density of 5000 cells/μL. When distilled water was used to prepare the cell suspension, the osmotic pressure damaged the cells. This experiment also employed distilled water to confirm whether the detection sensitivity was sufficient. The cell suspension with a volume of 1 μL was dropped onto an ITO-coated glass slide and dried in a vacuum. ATT/NaY was made by dissolving in water with 50% acetone at a concentration of 10 mg/mL, and dropped with a volume of 1 μL on the dried spot of the cell suspension. This corresponded to an ATT/NaY density of about 0.56 mg/cm^2^.

**Figure 3:**
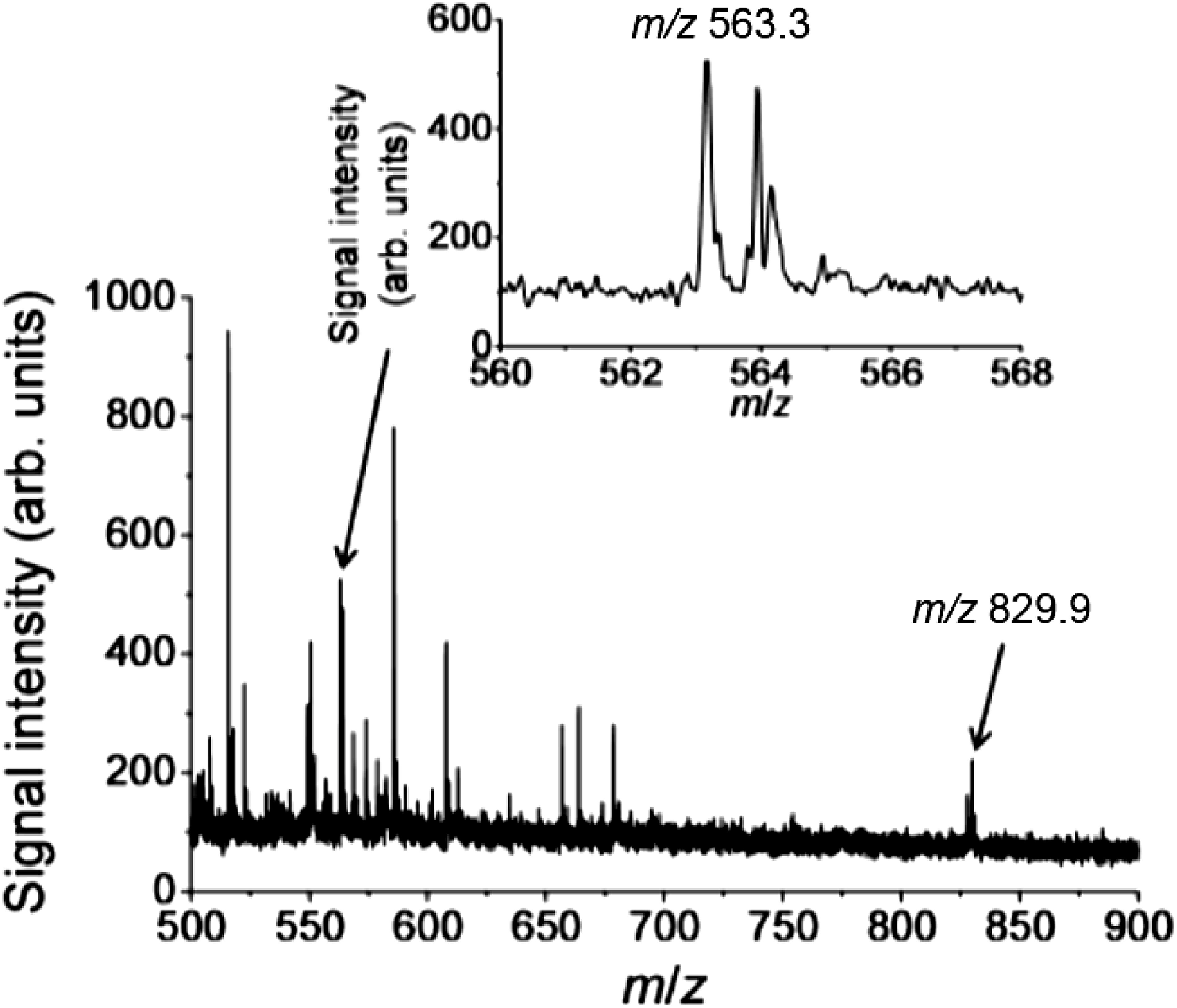
Fig. 3. Typical mass spectra obtained from a cell suspension of PC-3 when ATT/NaY was dropped.

Figure 3 shows typical mass spectra obtained from the cell suspension of PC-3 when the matrix was dropped. The ions at *m*/*z* 563.3 and 829.9 were detected from the cell suspension simultaneously. Hence, the zeolite matrix was effective to simultaneously detect PpIX and docetaxel administered in PC-3 cells.

### Comparison of the uptakes between PC-3 and PC-3-DR

To assess the uptakes of an anticancer drug and a photosensitizer in cancer cells, we compared the uptakes between PC-3 cells and PC-3-DR cells. PC-3 and PC-3-DR cells were cultured separately on ITO-coated glass slides for 24 h by 2.5×10^5^ cells/dish. Drugs were administered in the cells in a similar way as described above. As a control, PC-3 was incubated in a medium without drugs. After removing the medium, the cells were washed once with Dulbecco’s phosphate buffered saline followed by aqueous ammonium acetate solution at a concentration of 150 mM that was adjusted pH 7.4 using 1 M ammonium hydroxide. Ammonium acetate has been reported as an optimal medium for secondary ion mass spectrometry because less salt precipitates on the sample compared to other methods and a good mass spectrum can be obtained.^14)^ An ammonium acetate concentration of 150 mM and a pH of 7.4 were prepared to be isotonic with the intracellular fluid based on the extracellular concentration of general mammalian cells.^15)^ After the wash, the cells were immediately dried in a vacuum without the fixation process. The zeolite matrix was prepared under the same conditions as above and dropped onto the cells with a volume of 1 μL.

**Figure 4:**
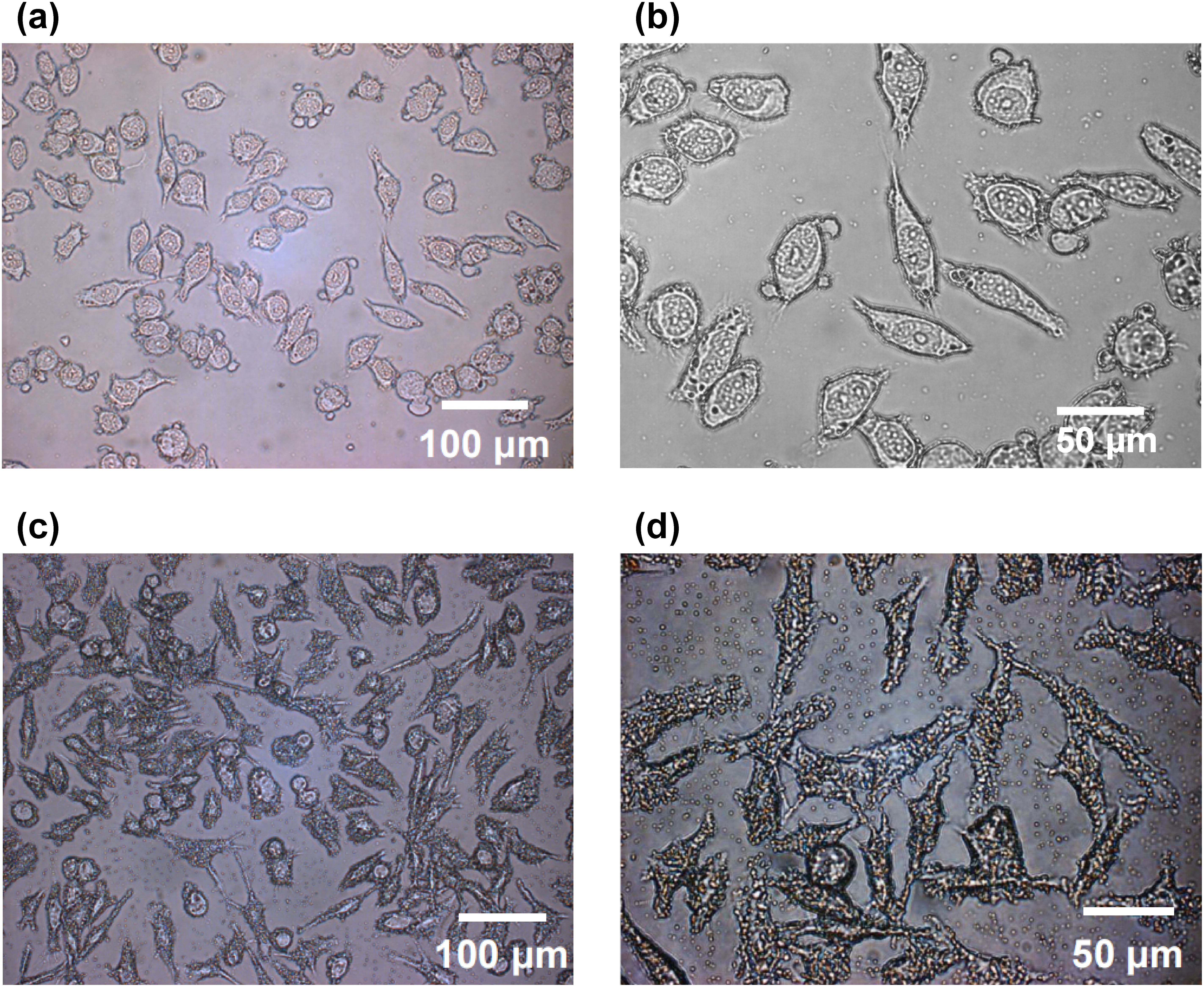
Fig. 4. (a) Bright-field image and (b) magnified image obtained from PC-3 cells washed by ammonium acetate. (c) Bright-field image and (d) magnified image obtained from PC-3 cells washed by D-PBS.

Figure 4 shows (a) a bright-field image and (b) a magnified image obtained from PC-3 cells washed by ammonium acetate. (c) Bright-field image and (d) magnified image were obtained from PC-3 cells washed by D-PBS. The cell morphology was preserved by washing with ammonium acetate (Fig. 4(a)) and D-PBS (Fig. 4(c)). However, more salt was precipitated when washed by D-PBS (Fig. 4(d)) than when washed by ammonium acetate (Fig. 4(c)). Therefore, it was presumed that ion suppression caused by salt can be reduced by washing with ammonium acetate.

**Figure 5:**
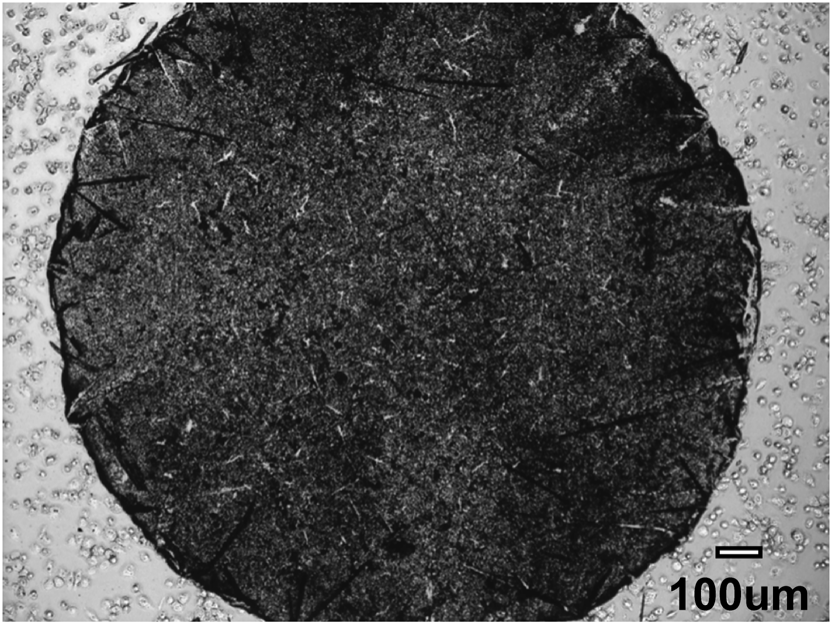
Fig. 5. A typical bright-field image obtained from the sample after the zeolite matrix was dropped on the cells.

Figure 5 shows a typical bright-field image obtained from the sample after the zeolite matrix was dropped on the cells. Cells were present in the entire image of the ITO-coated glass slide and in the area of the matrix dropped.

**Figure 6:**
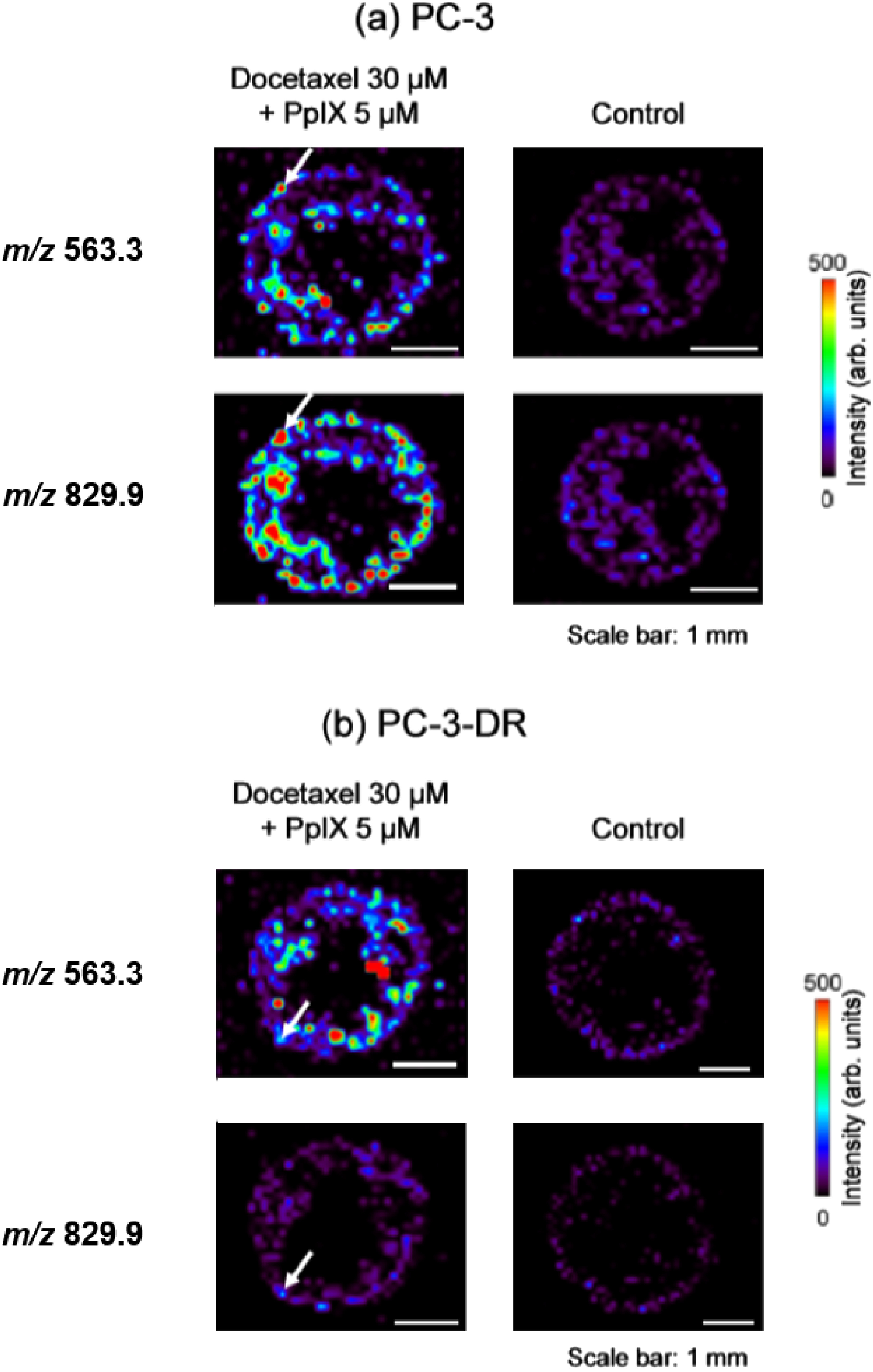
Fig. 6. Ion image in mass spectrometry obtained from (a) PC-3 and (b) PC-3-DR cells administered in 5 μM PpIX and 30 μM docetaxel when a zeolite matrix was dropped on the cells.

Figure 6 shows the mass spectrometry imaging results obtained from (a) PC-3 and (b) PC-3-DR cells administered in 5 μM PpIX and 30 μM docetaxel when the zeolite matrix was dropped on the cells. Ions at *m*/*z* 563.3 and 829.9 were detected simultaneously from the PC-3 cells. The ion at *m*/*z* 563.3 was also detected in PC-3-DR cells, but that at *m*/*z* 829.9 was not detected. Hence, PC-3-DR cells had docetaxel resistance. The accumulation of PpIX in PC-3 and PC-3-DR cells suggested the effectiveness of PDT for not only PC-3 but also PC-3-DR cells.

**Figure 7:**
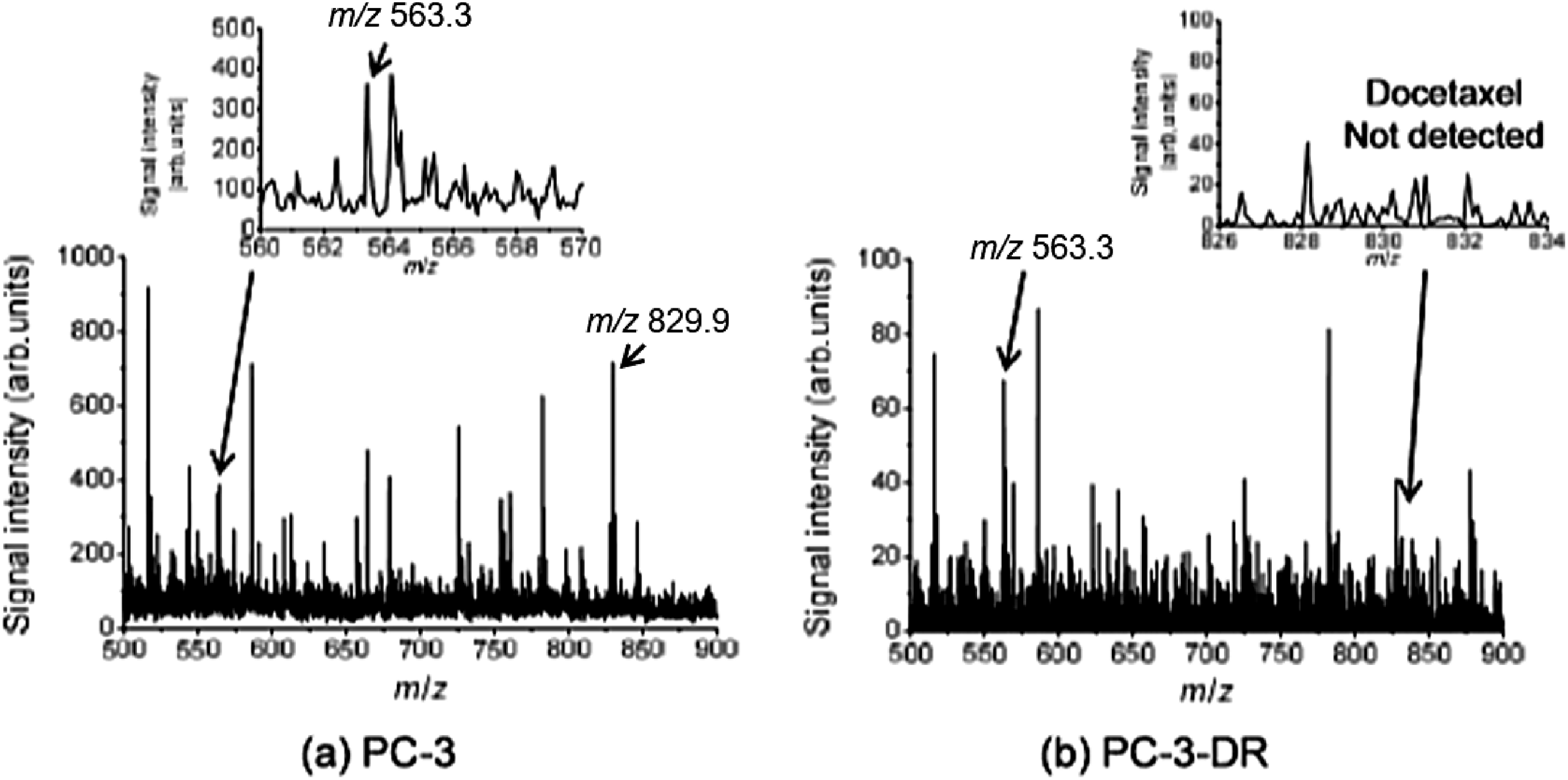
Fig. 7. Typical mass spectra obtained from (a) PC-3 and (b) PC-3-DR cells present at the point of the white arrow in Fig. 6.

Figure 7 shows typical mass spectra obtained from (a) PC-3 and (b) PC-3-DR cells, which existed at the point of the white arrow in Fig. 6. This point was about 100 μm square and corresponded to one laser irradiation point. The ions at *m*/*z* 563.3 and 829.9 were detected simultaneously from the PC-3 cells. However, only the ion at *m*/*z* 563.3 was detected from the PC-3-DR cells. The cell size was about several to tens of micrometers, and the laser diameter was about 100 μm. Thus, a single laser irradiation point spanned multiple cells. In the future, we will investigate detection of multiple drugs in a single cell. For the uptake evaluation of PpIX and docetaxel in a single cell, a higher spatial resolution MSI device^16,17)^ and fine matrix crystals^18–20)^ are necessary.

## CONCLUSION

We investigated the uptakes of the photosensitizer, PpIX, and the anticancer drug, docetaxel, in cancer cells. Since the ionization efficiency of docetaxel was too low for conventional MALDI-MS, a zeolite matrix was prepared by mixing a conventional organic matrix, ATT, and NaY5.6 zeolite. Upon spraying the zeolite matrix on a pure mixture of these drugs, PpIX and docetaxel were detected simultaneously. Thus, the zeolite matrix could simultaneously detect PpIX and docetaxel. As a next step, the detection of drugs administered in human prostate cancer cells PC-3 was examined using the zeolite matrix. Dropping the zeolite matrix on a cell suspension, PpIX and docetaxel were detected simultaneously, demonstrating that the zeolite matrix could also detect these drugs administered in cancer cells. To evaluate the uptakes of PpIX and docetaxel in PC-3 and PC-3-DR cells, we investigated the simultaneous detection with a sample preparation that retained its cell shape. The cell morphology was preserved by washing with ammonium acetate. With this sample preparation, PpIX and docetaxel were detected simultaneously from the PC-3 cells but only PpIX was detected from the PC-3-DR cells. Hence, we confirmed that PpIX was taken up by docetaxel-resistant cancer cells.

## Notes

Mass Spectrom (Tokyo) 2020; 9(1): A0091
